# Iron Sulfur and Molybdenum Cofactor Enzymes Regulate the *Drosophila* Life Cycle by Controlling Cell Metabolism

**DOI:** 10.3389/fphys.2018.00050

**Published:** 2018-02-14

**Authors:** Zvonimir Marelja, Silke Leimkühler, Fanis Missirlis

**Affiliations:** ^1^Imagine Institute, Université Paris Descartes—Sorbonne Paris Cité, Paris, France; ^2^Department of Molecular Enzymology, Institute of Biochemistry and Biology, University of Potsdam, Potsdam, Germany; ^3^Departamento de Fisiología, Biofísica y Neurociencias, Centro de Investigación y de Estudios Avanzados del Instituto Politécnico Nacional, Ciudad de México, Mexico

**Keywords:** aldehyde oxidase, DNA polymerase, electron transport chain, ecdysone, iron regulatory protein, quiescent mitochondria, magnetoreceptor, mitoflashes

## Abstract

Iron sulfur (Fe-S) clusters and the molybdenum cofactor (Moco) are present at enzyme sites, where the active metal facilitates electron transfer. Such enzyme systems are soluble in the mitochondrial matrix, cytosol and nucleus, or embedded in the inner mitochondrial membrane, but virtually absent from the cell secretory pathway. They are of ancient evolutionary origin supporting respiration, DNA replication, transcription, translation, the biosynthesis of steroids, heme, catabolism of purines, hydroxylation of xenobiotics, and cellular sulfur metabolism. Here, Fe-S cluster and Moco biosynthesis in *Drosophila melanogaster* is reviewed and the multiple biochemical and physiological functions of known Fe-S and Moco enzymes are described. We show that RNA interference of *Mocs3* disrupts Moco biosynthesis and the circadian clock. Fe-S-dependent mitochondrial respiration is discussed in the context of germ line and somatic development, stem cell differentiation and aging. The subcellular compartmentalization of the Fe-S and Moco assembly machinery components and their connections to iron sensing mechanisms and intermediary metabolism are emphasized. A biochemically active Fe-S core complex of heterologously expressed fly Nfs1, Isd11, IscU, and human frataxin is presented. Based on the recent demonstration that copper displaces the Fe-S cluster of yeast and human ferredoxin, an explanation for why high dietary copper leads to cytoplasmic iron deficiency in flies is proposed. Another proposal that exosomes contribute to the transport of xanthine dehydrogenase from peripheral tissues to the eye pigment cells is put forward, where the Vps16a subunit of the HOPS complex may have a specialized role in concentrating this enzyme within pigment granules. Finally, we formulate a hypothesis that (i) mitochondrial superoxide mobilizes iron from the Fe-S clusters in aconitase and succinate dehydrogenase; (ii) increased iron transiently displaces manganese on superoxide dismutase, which may function as a mitochondrial iron sensor since it is inactivated by iron; (iii) with the Krebs cycle thus disrupted, citrate is exported to the cytosol for fatty acid synthesis, while succinyl-CoA and the iron are used for heme biosynthesis; (iv) as iron is used for heme biosynthesis its concentration in the matrix drops allowing for manganese to reactivate superoxide dismutase and Fe-S cluster biosynthesis to reestablish the Krebs cycle.

## Introduction

In the first known biochemical reactions on earth, molybdenum and iron-sulfur (Fe-S) clusters enabled electron transfers turning inorganic molecules into hydrogenated carbon molecules (Mortenson, 1964; Eck and Dayhoff, 1966; Hall et al., 1971; Ochiai, 1978; Wächtershäuser, 1988; Russell and Martin, 2004; Zhang and Gladyshev, 2008; Nitschke and Russell, 2009; Schoepp-Cothenet et al., 2012; Stüeken et al., 2015). Similar biochemistry remains active in living organisms carried out by a variety of metallo-enzymes. In this *hypothesis and theory* article, we present examples of Fe-S and molybdenum cofactor (Moco) enzymes from the dipteran fly *Drosophila melanogaster*, a genetically amenable and thoroughly characterized experimental model system (Bellen et al., 2010; St Johnston, 2013; Mohr et al., 2014). By looking at their multiple physiological functions, we propose that Fe-S enzymes are central in the development, life cycle transitions and aging of flies. Given the conservation of these phenomena in the evolution of the animal kingdom, we anticipate that many of our descriptions will be transferable to other organisms.

In the first part of the article, we present how Fe-S clusters are formed, a process that has been studied extensively by biochemists in prokaryotes (Roche et al., 2013; Blanc et al., 2015), yeast (Martinez-Pastor et al., 2017), plants (Balk and Schaedler, 2014), and humans (Paul and Lill, 2015; Rouault and Maio, 2017), but also by the biomedical community intent to find a therapy for patients with Friedreich's ataxia, caused by reduced expression of the frataxin (FXN) gene (Campuzano et al., 1996). Other Fe-S proteins are also implicated in human disease (Rouault, 2012; Beilschmidt and Puccio, 2014; Isaya, 2014). In *D. melanogaster*, the pioneering work of Maria Moltó and co-workers has almost exclusively focused on the *Drosophila* frataxin homolog describing what goes wrong when Fe-S biosynthesis is disrupted in flies (reviewed in Mandilaras et al., 2013; Tang and Zhou, 2013b; Zhu et al., 2014; Calap-Quintana et al., 2017). Furthermore, we describe the biosynthesis of Moco (Rajagopalan, 1997; Mendel and Leimkühler, 2015; Leimkühler, 2017), whose basic structure has two sulfur atoms of the tricyclic pyranopterin molecule molybdopterin (MPT) coordinating the Mo atom (Rajagopalan et al., 1982). Work on Moco enzymes in *Drosophila* started in the fifties and Victoria Finnerty studied the Moco biosynthetic pathway during the last quarter of the twentieth century (Kamdar et al., 1997). Her research program used the molybdoenzyme xanthine dehydrogenase (Xdh) encoded by the *rosy* gene, whose activity is required for the formation of eye pigments for reasons that are still not fully resolved (Phillips and Forrest, 1980; Wiley and Forrest, 1981; Ferre et al., 1986; Keith et al., 1987; Hilliker et al., 1992), and therefore mutants affecting Moco biosynthesis have evident eye color phenotypes (Kamdar et al., 1997). We complement this review with new discoveries in the role of proteins involved in Fe-S cluster and Moco biosynthesis by showing the original data, which are not published elsewhere (Marelja, 2013), and with a new hypothesis to explain previous observations that dietary copper decreases iron storage in *Drosophila* (Poulson and Bowen, 1952; Bettedi et al., 2011).

In the second part of the article, we review studies of the *Drosophila* molybdoenzymes Xdh, aldehyde oxidase (Aox), sulfite oxidase (Suox). Special attention is paid to the problem of how Xdh, a cytosolic enzyme that requires two Fe-S clusters, Moco and flavin for activity (Hughes et al., 1992; Doyle et al., 1996), finds its way into pigment granules of the eye, the only enzyme with such cofactors known to reside in the endomembrane system (Reaume et al., 1989, 1991). Our hypothesis is that exosomes are involved in the process. The *fly* literature on mitochondrial Fe-S enzymes required for respiration and the biosynthesis of ecdysone, heme, and lipoate is summarized. The role of cytosolic and nuclear Fe-S enzymes in DNA replication, transcription and translation is also reviewed, followed by a brief discussion of the regulation of the Fe-S cluster of iron regulatory protein-1A (IRP-1A) and cellular iron sensing in *Drosophila*. We then move to the question of how mitochondria sense iron, where we present a new hypothesis suggesting that the mitochondrial superoxide dismutase (Sod2) is a possible mitochondrial iron sensor. Our model of mitochondrial iron sensing also explains the previously observed superoxide bursts in mitochondria (Wang et al., 2008) and the connection between mitochondrial Fe-S cluster biosynthesis and lipogenesis (Tong and Rouault, 2007). Last, we revisit the question of whether Fe-S and/or Moco enzymes are involved in the circadian clock (Mandilaras and Missirlis, 2012).

In the third part of the article, we communicate how profoundly cell physiology depends on Fe-S enzymes. We review the shifts in cell metabolism from glycolysis to aerobic respiration during development (Tennessen et al., 2011, 2014) and during stem cell differentiation (Homem et al., 2014; Sieber et al., 2016), emphasize the requirement of Fe-S clusters for growth through the larval stage and into metamorphosis (Anderson et al., 2005; Uhrigshardt et al., 2013; Llorens et al., 2015; Palandri et al., 2015) and the decline of mitochondrial respiration during aging (Vann and Webster, 1977; Yan et al., 1997; Ferguson et al., 2005). We then discuss the interesting finding that in the female germ line, stem cell differentiation requires the mitochondrial ATP synthase, but not the respiratory chain enzymes (Teixeira et al., 2015). We critically evaluate the possibility that the presence of quiescent mitochondria in the female germ line may serve as a protective hereditary mechanism against the accumulation of mutations in their genome (Allen, 1996). Finally, we describe specific functions of Fe-S or Moco enzymes in the major organs of the fly, concluding that they are a biochemically active component of the complex organization that characterizes living animals.

## Fe-S cluster and moco biosynthesis

**Figure 1:**
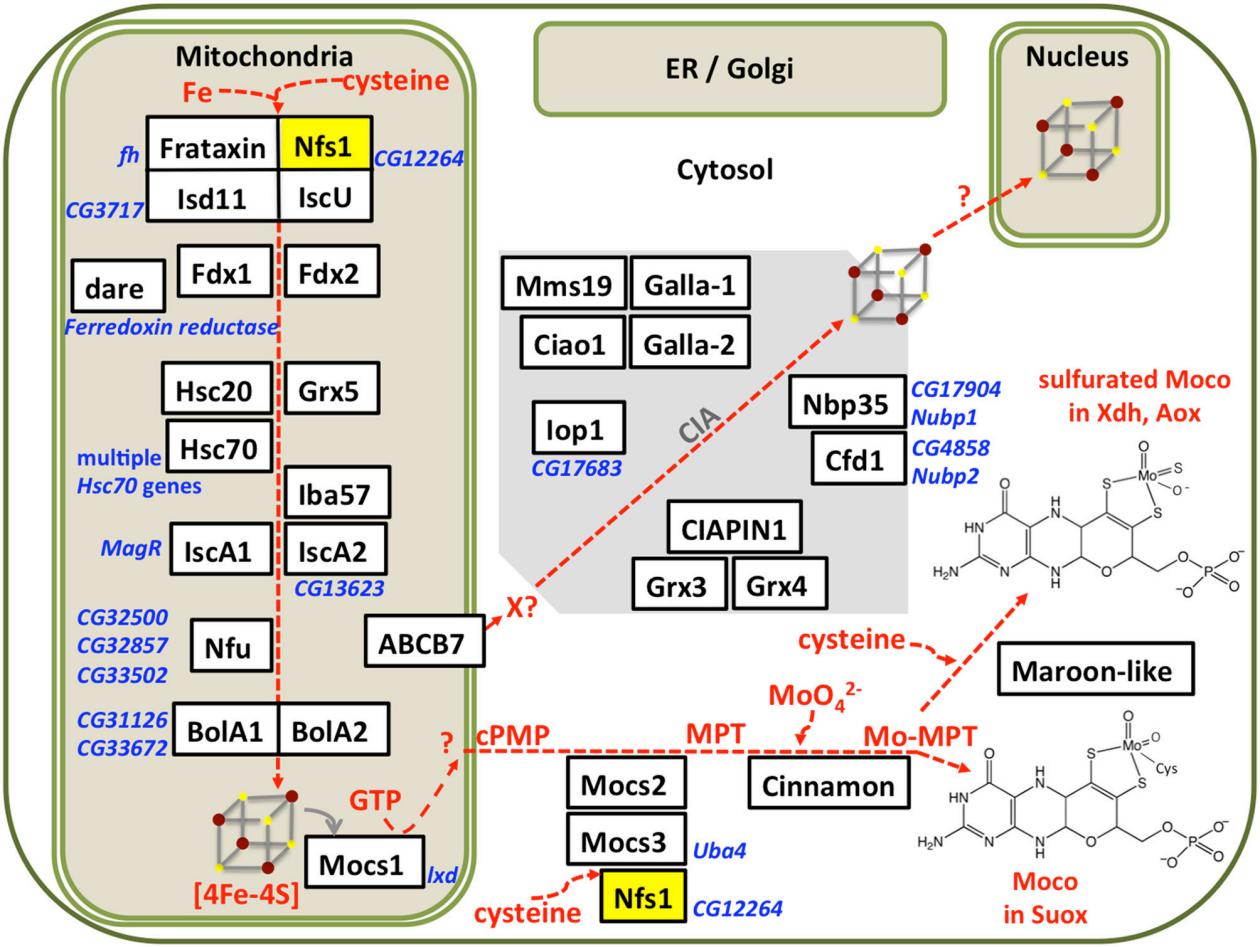
Subcellular compartmentalization of the Fe-S cluster and Moco assembly processes. Iron and sulfur from cysteine are assembled on IscU in a process that requires electrons provided by ferredoxins (Fdx1, Fdx2, which are themselves Fe-S proteins). Chaperones and Grx5 are required for transfer of the Fe-S cluster to further scaffold proteins. The *Drosophila* homologs were identified by Blast searches, proposed names for the gene products (in white boxes) were based on the corresponding nomenclature in humans, current Flybase annotations are shown in blue font. Our own results (Marelja, 2013) confirm that the *Drosophila* genes *CG12264* and *CG3717* are the homologs of the human genes NFS1 and ISD11, respectively. Multiple Fe-S enzymes are present in mitochondria, cytosol, and nucleus, but so far none have been reported in the secretory pathway. All known components of the CIA pathway are conserved in *Drosophila*, but the first steps of CIA remain to be elucidated. Moco biosynthesis initiates in the mitochondria with the Fe-S protein Mocs1 and continues in the cytosol with Mocs2, Mocs3, and Cinnamon producing Moco used in Suox. Maroon-like sulfurates Moco for Xdh and Aox. Mitochondrial and cytosolic forms of the cysteine desulfurase Nfs1 (yellow box) provide sulfide to Fe-S cluster and Moco biosynthesis, respectively.

Fe-S cluster assembly initiates in the mitochondrial matrix. We summarize the different steps of the assembly process (Figure 1); for detailed reviews of the pathway the reader is referred elsewhere (Roche et al., 2013; Balk and Schaedler, 2014; Blanc et al., 2015; Paul and Lill, 2015; Martinez-Pastor et al., 2017; Rouault and Maio, 2017). The mitoferrin transporter ensures mitochondrial iron uptake, whereas the L-cysteine desulfurase Nfs1 provides the inorganic sulfide as persulfide. Electrons are required for the cleavage of the persulfide group and assembly of the cluster, which are supplied from ferredoxins Fdx1 and Fdx2. The first assembly protein is IscU, which can accommodate [2Fe-2S] or [4Fe-4S] clusters. Fe-S clusters are transferred from IscU to other scaffold proteins like IscA1, IscA2, Iba57, Nfu, BolA1, or BolA2 that show specificity of cluster delivery to the target enzymes. For Fe-S cluster transfer between the assembly proteins, or between assembly proteins and target enzymes, specialized chaperones (Hsc20 and its cognate partner) and glutaredoxin-5 (Grx5) are required. A cytosolic Fe-S cluster assembly (CIA) pathway has been described, however it is unclear how the first clusters are formed on the CIA complex. The cytosolic electron donors cytokine-induced apoptosis inhibitor-1 (CIAPIN1) and glutaredoxins Grx3 and Grx4 have been identified (Figure 1). Only three genes involved in Fe-S biosynthesis have been individually studied in flies: (i) *Drosophila frataxin* whose exact biochemical function is still unclear (Cañizares et al., 2000; Anderson et al., 2005, 2008; Llorens et al., 2007; Kondapalli et al., 2008; Runko et al., 2008; Navarro et al., 2010, 2011, 2015; Shidara and Hollenbeck, 2010; Soriano et al., 2013, 2016; Tricoire et al., 2014; Calap-Quintana et al., 2015; Palandri et al., 2015; Chen et al., 2016b; Edenharter et al., 2017), (ii) *IscU* that encodes a protein assembly platform for Fe-S cluster biosynthesis (Dzul et al., 2017), and iii) *Hsc20* encoding one of two chaperones that mobilize the Fe-S cluster from IscU to downstream Fe-S proteins in the mitochondria (Uhrigshardt et al., 2013).

### The *Drosophila* Nfs1/Isd11/IscU/frataxin complex

**Figure 2:**
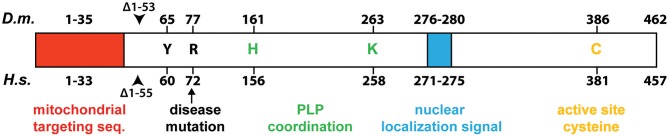
Schematic comparison of *Drosophila* Nfs1 and human NFS1. Highly conserved residues that play a role for desulfurase catalysis and disease are indicated: the active site cysteine (C) that forms the persulfide intermediate (Zheng et al., 1994; Lauhon et al., 2004); the tyrosine (Y) was shown to be crucial for activity in *Saccharomyces cerevisiae* Nfs1p (Mühlenhoff et al., 2004); the point mutation p.Arg72Gln (arrow) in human NFS1 was identified to cause infantile mitochondrial complex II/III deficiency (Farhan et al., 2014); whereas histidine (H) and lyine (K) are involved in pyridoxal 5′-phosphate (PLP) coordination (Cupp-Vickery et al., 2003). The proposed N-terminal mitochondrial targeting sequences is shown by a *red box*, while the nuclear localization signal is shown as a *blue box*. The beginning of the truncated Nfs1/NFS1 versions used for biochemical purification are indicated by an arrow head (Marelja, 2013). Numbers indicate amino acid position in the *Homo sapiens* (*H.s*.) and *D. melanogaster* (*D.m*.) proteins.

Two *Drosophila* Fe-S cluster assembly proteins have been purified to date, frataxin (Kondapalli et al., 2008) and IscU (Dzul et al., 2017). We characterized the core mitochondrial protein complex involved in Fe-S cluster assembly in flies (Marelja, 2013). Based on previous work with the human L-cysteine desulfurase NFS1 (Marelja et al., 2008, 2013), the *Drosophila* Nfs1 homolog (CG12264) was purified. The human and *Drosophila* enzymes share an amino acid sequence identity of 78% when the mitochondrial targeting sequences are removed from the calculation. All amino acids implicated in enzymatic function of the human protein are conserved in *Drosophila* Nfs1 (Figure 2).

**Figure 3:**
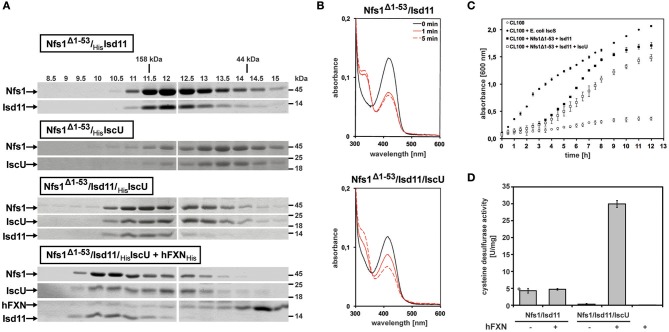
*In vitro* characterization of *Drosophila* Nfs1, Isd11, IscU in complex with human frataxin. **(A)** Co-expression of Nfs1^Δ1–53^ with Isd11 and/or IscU in *Escherichia coli*. After purification of the complexes with affinity chromatography, 30 μM of Nfs1^Δ1–53^ was applied onto the analytical Superdex200 size exclusion column and compared by SDS-PAGE. The fractions show that the Nfs1^Δ1–53^/Isd11 complex eluted earlier than the IscU/Nfs1^Δ1–53^ complex, indicating its smaller size. As expected, the complex between all three proteins was larger. Human frataxin added to the purified Nfs1^Δ1–53^/Isd11/IscU complex also bound as demonstrated by further increase in size. Gamma globulin (158 kDa) and ovalbumin (44 kDa) were standards (kDa ladder on the top; fractions in ml units). **(B)** UV-Vis absorption spectra of 10 μM Nfs1^Δ1–53^ in complex with His_6_-Isd11 (top panel) and Isd11/His_6_-IscU (bottom panel) in the absence (*solid black line*) or 1 min and 5 min after addition of 4 mM L-cysteine (*red lines*). All spectra were recorded at 23°C in 500 μl 100 mM Tris (pH 8.0), 200 mM NaCl, 1 mM EDTA. **(C)** Growth curves of 100 ml cultures of the *Escherichia coli* CL100 strain (Δ*iscS*) transformed either with vectors pET15b and pACYCDuet-1 or with plasmids containing coding sequences for *Drosophila* Nfs1^Δ1–53^, Isd11, IscU, or *Escherichia coli* IscS were recorded after addition of 100 μM IPTG for 12h at 30°C. **(D)** Desulfurase activity of Nfs1^Δ1–53^/Isd11 and Nfs1^Δ1–53^/Isd11/IscU complexes in absence or presence of human FXN. L-cysteine desulfurase activity was measured by determination of total sulfide produced. One unit is defined as the amount of enzyme producing 1 μmol of sulfide/min. Under anaerobic conditions, Nfs1^Δ1–53^/Isd11 and Nfs1^Δ1–53^/Isd11/IscU complexes were mixed in a 1:3 [Nfs1]:[FXN] ratio and incubated for 10 min at 23°C. The last lane (hFXN +) is a negative control to show that the human FXN preparation does not carry contaminant desulfurase activity. Error bars indicate the standard deviation of three measurements.

The first 53 amino acids of the full length *Drosophila* Nfs1 were removed from an *Escherichia coli*-driven protein expression construct (for detailed Materials and Methods see Marelja, 2013). Nfs1^Δ1–53^ was co-expressed with *Drosophila* Isd11 (CG3717 shows 44% amino acid sequence identity to human ISD11; Adam et al., 2006; Wiedemann et al., 2006) or with *Drosophila* IscU (Dzul et al., 2017), the scaffold protein where newly formed Fe-S clusters are initially assembled (Zheng et al., 1998; Gerber et al., 2003; Fox et al., 2015; Parent et al., 2015). The respective Nfs1^Δ1–53^/Isd11 and Nfs1^Δ1–53^/IscU complexes were affinity purified and recovered in a stable, soluble form. Higher molecular weight complexes were obtained when all three *Drosophila* proteins were co-expressed and also when human FXN (a gift from Kuanyu Li; Xia et al., 2012; Friemel et al., 2017) was added to the Nfs1^Δ1–53^/Isd11/IscU complex (Figure 3A). Our own efforts to purify *Drosophila* frataxin as in Kondapalli et al. (2008) were unsuccessful (Marelja, 2013).

The characteristic yellow color observed for other L-cysteine desulfurases containing pyridoxal 5′-phosphate (PLP) as a prosthetic group was confirmed in absorption spectra of *Drosophila* Nfs1 at 420 nm (Figure 3B, solid black lines on both panels). Moreover, addition of the enzyme's substrate L-cysteine induced a decrease of absorbance at 420 nm and an increase of absorbance at 320 nm (Figure 3B, solid and dashed red lines), as reported for *Azotobacter vineladii* L-cysteine desulfurase NifS, showing that the α-amino group of cysteine binds to the PLP at the enzyme's active site (Zheng et al., 1993). For Nfs1^Δ1–53^/Isd11, L-cysteine binding reached equilibrium at 1 min (Figure 3B, top panel). In contrast, binding of L-cysteine to the Nfs1^Δ1–53^/Isd11/IscU complex was slower (Figure 3B, bottom panel), suggesting that the presence of IscU reduced substrate accessibility to Nfs1 *in vitro*. The *Drosophila* Nfs1^Δ1–53^/Isd11 complex was also investigated for its ability to restore the growth deficiency of the *E. coli* Δ*iscS* strain CL100 (Figure 3C). As expected, CL100 transformed with the plasmid containing the endogenous *iscS* gene restored the growth defect. *Drosophila* Nfs1^Δ1–53^/Isd11 partially complemented IscS function in the *E. coli* CL100 strain, whereas expression of IscU along with Nfs1^Δ1–53^/Isd11 slowed down bacterial growth (Figure 3C). Notably, this *in vivo* result matches the *in vitro* observation that L-cysteine binding to Nfs1^Δ1–53^ is slower in the presence of Isd11 and IscU (Figure 3B). These findings are consistent with the previous demonstration that the L-cysteine desulfurase activity of the human NFS1/ISD11 complex is reduced in the presence of ISCU (Tsai and Barondeau, 2010; Bridwell-Rabb et al., 2014).

To further test this notion, we tested whether IscU altered the desulfurase activity of purified Nfs1 *in vitro* (Figure 3D). Sulfide production activity was determined with methylene blue in the presence of 1,4 dithiothreitol (Fogo and Popowsky, 1949; Urbina et al., 2001). Only weak activity was detected from the purified Nfs1^Δ1–53^/Isd11 complex, possibly because the complex was unable to perform reaction turnovers. Addition of IscU, forming the IscU/Nfs1^Δ1–53^/Isd11 complex, abolished sulfide production, consistent with observations presented above. Addition of human frataxin to the ternary IscU*/*Nfs1^Δ1–53^/Isd11 complex led to a six-fold increase in sulfide production activity, showing that frataxin stimulated the desulfurase activity of Nfs1/Isd11 in an IscU-dependent manner (Figure 3D). Steady-state kinetic parameters were determined by varying L-cysteine concentration for Nfs1^Δ1–53^ activity (after Nfs1^Δ1–53^/Isd11/IscU/frataxin complex formation) showing a catalytic efficiency of 149 M^−1^ s^−1^, k_cat_ 2.6 min^−1^, and a K_M_ of 0.290 mM. Taken together, these data indicate that Isd11, IscU, and frataxin are required for the activation of Nfs1 in *Drosophila*, providing experimental evidence that the initial complex of the mitochondrial Fe-S cluster biosynthetic machinery is conserved in *Drosophila* similar to what is known in other eukaryotes, including species with non-respiring mitochondria (Tovar et al., 2003; Richards and van der Giezen, 2006).

### Fe-S cluster biosynthesis occurs in distinct cellular compartments

**Figure 4:**
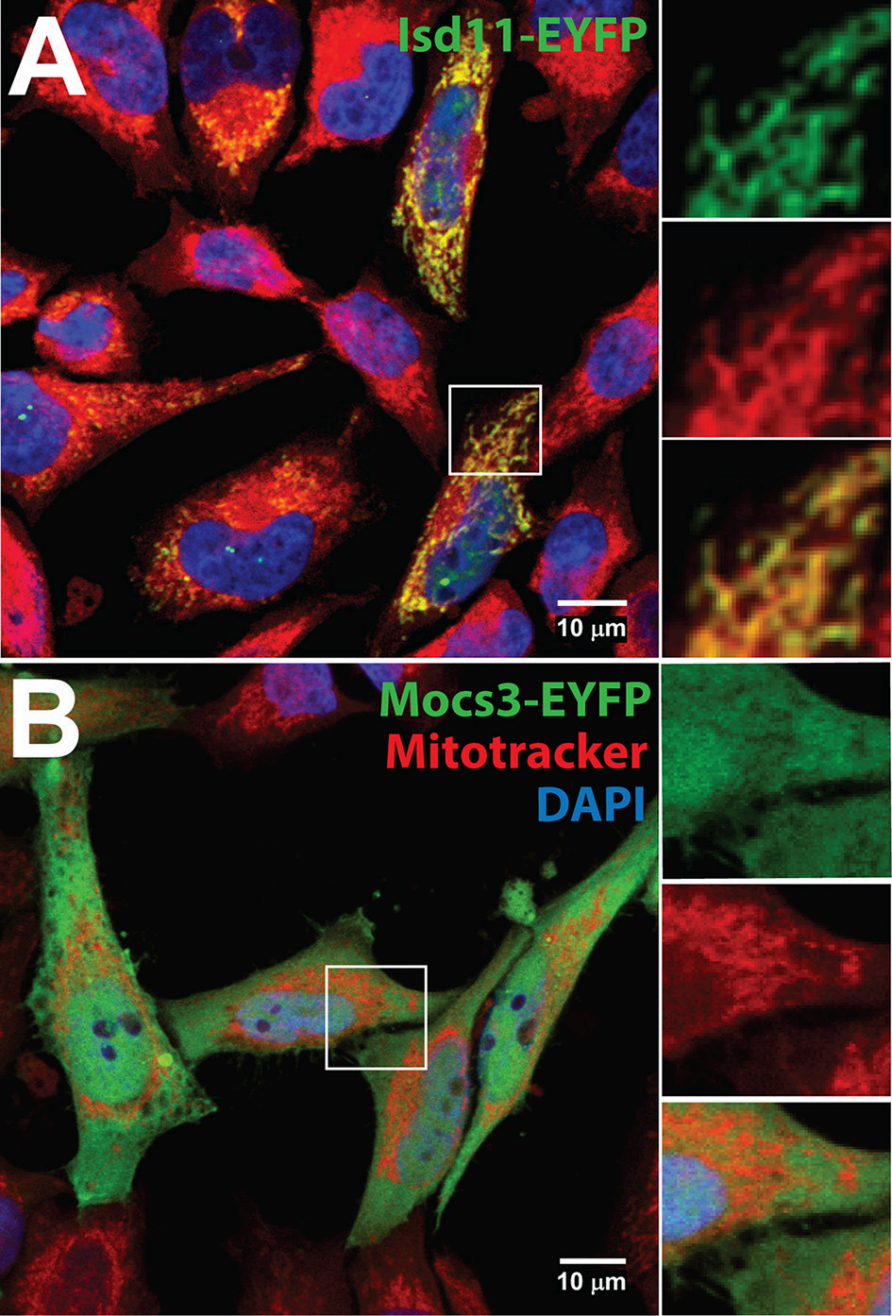
Subcellular localization of Isd11 and Mocs3. Confocal microscopy of **(A)** Hela cells transfected with a C-terminal fusion of EYFP to Isd11, stained with Mitotracker Red to detect mitochondria and with DAPI to visualize nuclei. Insets show two channels separately to appreciate co-localization of the fluorescent signals from Isd11-EYFP and Mitotracker (merged, bottom inset); **(B)** Hela cells transfected with Mocs3-EYFP, which localizes in the cytosol and nuclei.

Like the other proteins of the core complex described above, *Drosophila* Isd11 is localized in mitochondria (Figure 4A). Two mitochondrial ferredoxins (Palandri et al., 2015) and the *Drosophila* homolog of ferredoxin reductase (also known as adrenodoxin reductase encoded by the *dare* gene; Freeman et al., 1999) are required as electron donors for Fe-S cluster formation (Cai et al., 2017). Mitochondrial monothiol glutaredoxin-5 (CG14407) has not been investigated in *Drosophila*, but is likely required for Fe-S cluster biosynthesis (Rodríguez-Manzaneque et al., 2002; Wingert et al., 2005; Ye et al., 2010; Johansson et al., 2011), participating in the Fe-S cluster transfer to BolA-like proteins (Aldea et al., 1988; Frey et al., 2016; Melber et al., 2016; Uzarska et al., 2016; Nasta et al., 2017). Fe-S clusters are also transferred to Nfu-like (Tong et al., 2003; Melber et al., 2016; Wachnowsky et al., 2016) or Isa-like proteins (Jensen and Culotta, 2000; Kaut et al., 2000; Muhlenhoff et al., 2011; Sheftel et al., 2012) with the chaperone activity of mitochondrial Hsc20 (Uhrigshardt et al., 2010; Sieber et al., 2016) and its cognate Hsc70 [not clear which of several candidate *Hsc70* genes present in the fly genome (Adams et al., 2000) functions in Fe-S cluster biosynthesis]. These scaffold proteins deliver the Fe-S clusters to target mitochondrial Fe-S enzymes.

Fe-S clusters are also assembled on proteins in the cytosol (Figure 1). Early work in this area suggested that core mitochondrial Fe-S cluster assembly proteins were also being directed to the cytosol (Land and Rouault, 1998; Tong and Rouault, 2000, 2006) and protein targeting sequences do not typically result in a unique destination for most proteins (Hegde and Bernstein, 2006). Furthermore, another set of cytosolic Fe-S cluster assembly (CIA) proteins has been described (reviewed in Roy et al., 2003; Hausmann et al., 2005; Paul and Lill, 2015). Briefly, an Fe-S cluster is assembled on CFD1 and NBP35 with electron donors provided by CIAPIN1. The clusters are transferred through IOP1 to a scaffold with CIA1, CIA2, and target proteins. These CIA proteins are conserved in *Drosophila* (Figure 1), but little work exists in the context of Fe-S cluster assembly. The fly Ciao1 homolog was shown to be required for viability (Radford et al., 2005) prior to assigning its biochemical function as part of the Fe-S scaffold complex in CIA (Balk et al., 2005a). Ciao1 received its name from the word “bridge” in the Chinese language (Johnstone et al., 1998), whereas the yeast homolog was conveniently designated Cia1 when its interaction with the hydrogenase-like Nar1 (IOP1 in the human nomenclature; Huang et al., 2007) was discovered (Balk et al., 2005b). The fly CIA2 scaffold (Zhao et al., 2015; Vo et al., 2017) homologs Galla-1 and Galla-2 (Yeom et al., 2015) associate with target nuclear Fe-S proteins Mms19 (Gari et al., 2012; Papatriantafyllou, 2012; Stehling et al., 2012; Nag et al., 2018) and Xeroderma pigmentosum D (Xpd) (Rudolf et al., 2006). The *Drosophila* CIAPIN1 homolog is required for oogenesis (Marzuk et al., 2013). CIAPIN1 carries an Fe-S cluster (Zhang et al., 2008) and receives electrons from a cytosolic reductase (Netz et al., 2010). Neither the reductase nor cytosolic monothiol glutaredoxins (Li et al., 2009; Mühlenhoff et al., 2010; Banci et al., 2015) have been characterized in *Drosophila*. Furthermore, it is important to stress that no experimental data exist to implicate the *Drosophila* proteins Cfd1, Ciao1, CIAPIN1, Galla-1, Galla-2, Iop1 Mms19, Nbp35 in the assembly of cytosolic Fe-S clusters; there inclusion here and elsewhere (Mandilaras and Missirlis, 2012) as members of the CIA was exclusively based on gene homology searches. Nevertheless, many projects are being undertaken in other systems to better describe the process of Fe-S cluster assembly in cytosol and nucleus and we hope to see contributions from studies in *Drosophila* in the near future.

Finally, it is noteworthy that no Fe-S enzymes have been reported in the secretory pathway, implying that the presence of thiol-reducing activity in the cytosol, nucleus and mitochondrial matrix is essential for their stability. In insects, such activity rests on the thioredoxin reductase system (Kanzok et al., 2001; Missirlis et al., 2001, 2002).

### Excess copper inhibits Fe-S cluster biosynthesis

Copper and iron homeostasis are intimately linked (Fox, 2003). Physiologically, the two metals are required for aerobic respiration, albeit as separate cofactors (Villee, 1948). Poulson and Bowen made an early observation that when *Drosophila* larvae were fed a diet rich in copper their iron stores were being depleted (Poulson and Bowen, 1952). A key way in which the two metals depend on each other was uncovered when ceruloplasmin and other multicopper oxidases (MCOs) were shown to act as ferroxidases (Osaki et al., 1966) and their activity was linked to iron trafficking across membranes (reviewed in Kosman, 2010). *Drosophila* MCOs are linked to iron homeostasis in ways that are still not understood (Bettedi et al., 2011; Lang et al., 2012; Peng et al., 2015), but *MCO3* mutants fed on copper also show a reduction in ferritin iron content (Bettedi et al., 2011). It was therefore important to identify another hypothesis to explain copper-mediated cellular iron deficiency. The discovery that excess copper displaces the Fe-S cluster of mitochondrial ferredoxin (Vallières et al., 2017), leading to disrupted CIA (Alhebshi et al., 2012), suggests that copper toxicity may directly inhibit Fe-S cluster biosynthesis also in *Drosophila*. Reducing Fe-S cluster biosynthesis in *Drosophila* with either RNA interference (RNAi) of *frataxin* or loss-of-function *Hsc20* mutants lead to mitochondrial iron accumulation and reduced ferritin expression (Anderson et al., 2005; Uhrigshardt et al., 2013; Navarro et al., 2015), offering a testable potential explanation of why excess dietary copper affects ferritin iron accumulation. In this respect, we also note that copper chelation ameliorated a fly model of Friedreich's ataxia (Soriano et al., 2016) and that the dithiol *Drosophila* glutaredoxin-1 was implicated in copper homeostasis (Mercer and Burke, 2016).

### A mitochondrial iron sulfur enzyme initiates moco biosynthesis

Turning to Moco biosynthesis, an ancient, ubiquitous and highly conserved pathway underpinning molybdenum biochemistry (Rajagopalan, 1997; Mendel and Leimkühler, 2015; Leimkühler, 2017), it can be divided into three major steps: (i) GTP is converted to cPMP, (ii) cPMP is converted to MPT by generation of the dithiolene group, and (iii) molybdate is then ligated to MPT forming Moco (Figure 1). Three fly genes involved in Moco biosynthesis have attracted individual attention: (i) *Mocs1* (formerly known as *low xanthine dehydrogenase, lxd*) encoding a mitochondrial Fe-S enzyme that converts 5′-guanosine triphosphate (GTP) to cyclic pyranopterin monophosphate (cPMP) (Keller and Glassman, 1964; Courtright, 1975; Duke et al., 1975; Bogaart and Bernini, 1981; Schott et al., 1986; Ho et al., 1992; Tahoe et al., 2002), (ii) *cinnamon* (*cin*) encoding a gephyrin homolog that inserts molybdate into MPT (Baker, 1973; Browder and Williamson, 1976; Kamdar et al., 1994; Wittle et al., 1999), and (iii) *maroon-like (mal)* that encodes a Moco sulfurase (Mitchell and Glassman, 1959; Hubby and Forrest, 1960; Finnerty et al., 1970; Marsh and Wieschaus, 1977; Kamleh et al., 2009).

**Figure 5:**
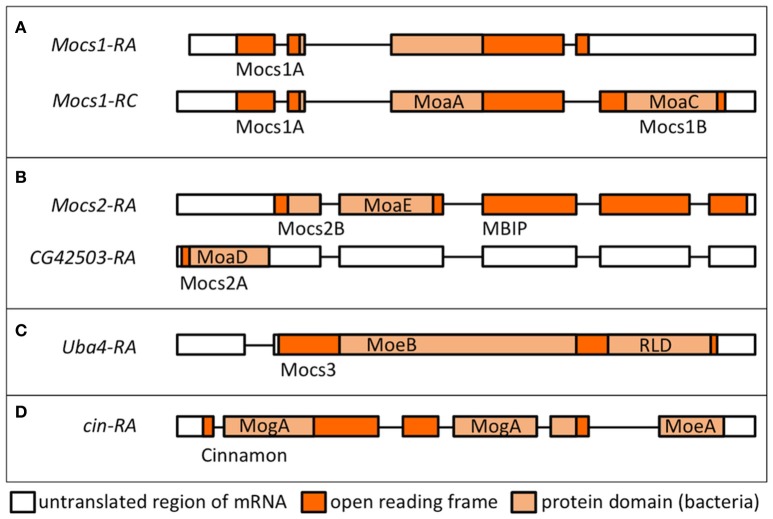
Transcriptional units and proteins encoded by *Drosophila* genes involved in Moco biosynthesis. Transcript names are shown as per Flybase. Protein domains in light orange boxes are according to the nomenclature in bacteria (Leimkühler, 2017). Names of the *Drosophila* proteins as used in text are shown below each transcript. **(A)** Alternative splicing of *Mocs1* results in short (Mocs1A) and long (Mocs1A-Mocs1B) subunits, which complex together to form the active enzyme. **(B)** A bicistronic transcript results in Mocs2B (which also carries the unrelated to Moco biosynthesis MBIP domain, see text) and Mocs2A. **(C)** Mocs3 carries the MoeB-like domain and a rhodanese-like domain (RLD), shown to interact with Nfs1 (Marelja et al., 2013). **(D)** Cinnamon carries the MogA/Gephyrin G domain at the N-terminus prior to the C-terminal MoeA/Gephyrin E domain (in reverse order to the mammalian Gephyrins).

Moco biosynthesis starts within mitochondria with a complex rearrangement reaction in which the C8 atom of the GTP purine is inserted between the 2′ and 3′-ribose carbon atoms (Wuebbens and Rajagopalan, 1993; Hover and Yokoyama, 2015). The human *MOCS1* gene is orthologous to the *lxd* locus (now renamed *Mocs1*) of *D. melanogaster* (Gray and Nicholls, 2000). Mutations in *lxd* affect molybdoenzyme activity in flies (Keller and Glassman, 1964). Alternative splicing at the *Mocs1* locus results in short (Mocs1A) and full length (Mocs1A-Mocs1B) proteins (Figure 5A). Mocs1A belongs to the superfamily of SAM-dependent radical enzymes (Hänzelmann and Schindelin, 2004), requiring a [4Fe-4S] cluster for the formation of a substrate radical by reductive cleavage of SAM. Studies on the human and bacterial homologs showed that Mocs1B participates in pyrophosphate cleavage after the formation of the 3′, 8cH_2_GTP intermediate (Hänzelmann and Schindelin, 2006). Evidence was sought for Mocs1 having a role in lifespan determination of *Drosophila*, as polymorphisms in the gene were detected between short- and long-lived inbred lines, but the results were inconclusive as the polymorphisms could not be associated with clear effects on enzyme activity (Tahoe et al., 2002). Mutants in *Mocs1* showed differential sensitivity to dietary molybdate compared to wild type strains (Duke et al., 1975).

### Molybdenum cofactor biosynthesis in the cytosol

**Figure 6:**
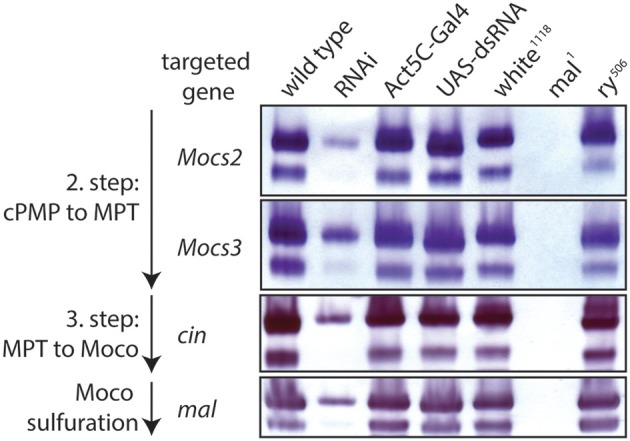
RNAi of Moco biosynthesis genes disrupts Aox activity. In-gel Aox activity is shown as described before (Marelja et al., 2014). Extracts from wild type flies are shown in column 1 of the gels, followed by extracts from RNAi of, respectively, *Mocs2, Mocs3, cinnamon*, and *maroon-like* in column 2 (note reduced Aox activity, consistent with a requirement of these genes for Moco biosynthesis). Columns 3 and 4 are extracts from the parental flies of the Gal4/UAS system that show normal Aox activity (controls), as do extracts from *white* and *rosy* mutants in columns 5 and 7 (positive controls), whereas extracts from the *mal* mutant show no detectable Aox activity (negative control, column 6).

In the second step, two sulfur atoms are transferred to cPMP to form MPT. This reaction is catalyzed by the heterotetrameric MPT synthase, which is composed of two small Mocs2A and two large Mocs2B subunits encoded from a single locus (Stallmeyer et al., 1999; Leimkuhler et al., 2003; Figure 5B). The physiological sulfur donor for MPT synthesis is Mocs3 (Matthies et al., 2004, 2005), which resides in the cytosol (Figure 4B). *Drosophila* Mocs2 and Mocs3 are both required for Aox activity (Figure 6). Based on what is known in human cells (Marelja et al., 2008), Mocs3 is expected to receive sulfur from Nfs1, the same protein that acts as a sulfur donor for Fe-S cluster biosynthesis in mitochondria, to which it binds through a rhodanese-like domain (RLD; Figure 5C). Using Förster resonance energy transfer and a split-EGFP system, NFS1 was shown to interact in the cytosol of human cells with MOCS3 (Marelja et al., 2013). This result was corroborated by showing that human NFS1/ISD11 requires MOCS3 to complement Moco biosynthesis in the *E. coli* deletion strain used in Figure 3C (Bühning et al., 2017).

Exceptionally, the *Drosophila* MPT synthase is linked to c-Jun N-terminal kinase (JNK) signaling, since Mocs2B forms a fusion protein with the mitogen activated protein kinase upstream binding inhibitory protein (MBIP; Figure 5B), which is one of 13 subunits of the Ada Two A containing histone acetyltransferase complex (ATAC) transcriptional co-activator (Suganuma et al., 2010). In human cells, Mocs2B/MBIP were shown to regulate ferritin translation through inhibition of PKR, a double-stranded RNA-dependent protein kinase (Suganuma et al., 2012, 2016). Whether this form of regulating iron storage is conserved in *Drosophila*, whether it serves to regulate the availability of Fe-S clusters for Mocs1, and whether *Drosophila* Nfs1 has a cytosolic function are questions for future research.

In the last step, molybdate is ligated to the dithiolene group of MPT producing Moco (Figure 1). The first results for the enzyme inserting molybdate to the pterin structure came from the Xdh deficiency of the *cin* mutation in *Drosophila* (Baker, 1973). Cin is partially homologous to the two *E. coli* proteins MogA and MoeA, which are also found as domains G and E of the rat protein Gephyrin, albeit in reverse orientation to the *Drosophila* protein (Kamdar et al., 1994; Stallmeyer et al., 1995; Feng et al., 1998; Figure 5D). The MogA-like G domain binds MPT and catalyzes the MPT-adenylation from Mg-ATP; MPT-AMP is then transferred to the MoeA-like E domain for hydrolysis and molybdenum insertion (Kuper et al., 2000, 2004; Schwarz et al., 2000; Llamas et al., 2004, 2006). Furthermore, the central domain in Gephyrin binds and anchors inhibitory ligand-gated anion channels in the postsynaptic membrane of neurons (Feng et al., 1998; Stallmeyer et al., 1999). However, it is unknown whether this additional function is conserved for *Drosophila* Cin.

**Figure 7:**
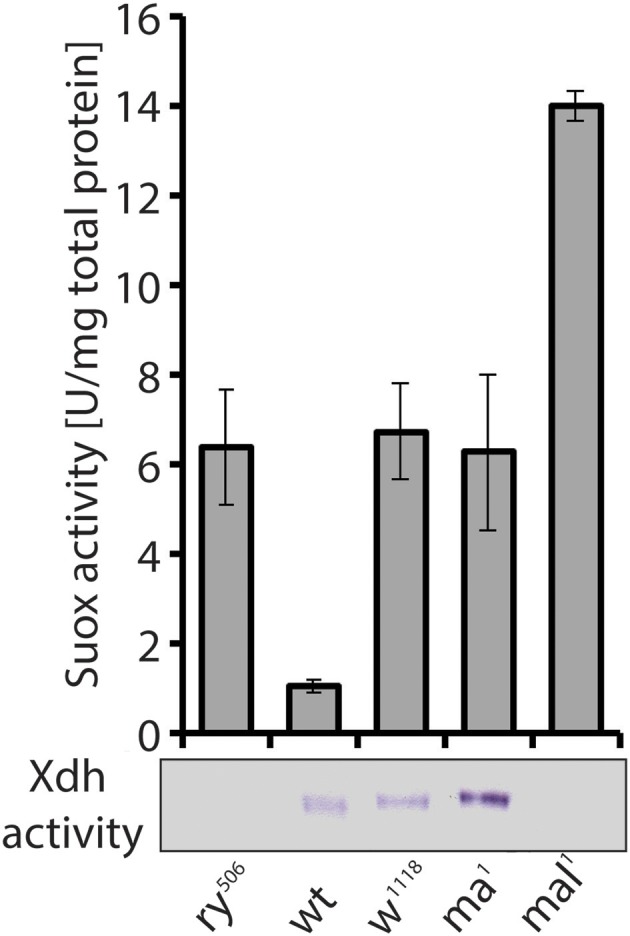
Despite similar eye pigment phenotypes with *rosy* and *mal*, the *maroon* mutant shows increased Xdh activity. Suox activity measured in extracts from mutants (*ry, rosy; w, white; ma, maroon; mal, maroon-like*) and a wild type (*wt*) strain is shown in bars. In-gel Xdh activity of the same extracts is also presented. Loss of Xdh activity in *rosy* and *mal* explains the phenotypic changes in eye pigmentation, but it was surprising to find increased Xdh activity in *maroon* mutants, given their eye phenotype.

After molybdenum insertion into MPT, Moco is either inserted into Suox or further modified by exchanging an oxo ligand by a sulfido group (Hille, 1996, 2002; Hille et al., 2014). The sulfur incorporation was also first discovered in the fly *mal* mutant, which lacked Xdh and Aox activities (Hadorn and Mitchell, 1951; Glassman and Mitchell, 1959; Hubby and Forrest, 1960; Forrest et al., 1961; Courtright, 1967) but retained or induced Suox activity (Figure 7) and an apparently normal concentration of total Moco (Bogaart and Bernini, 1981; Warner and Finnerty, 1981; Wahl et al., 1982). *In vitro* reconstitution of Xdh and Aox activities with sulfide/dithionite treatment led to the suggestion that *mal* affected the sulfur modification after the Mo insertion (Wahl and Rajagopalan, 1982; Wahl et al., 1982). A sequence comparison with the L-cysteine desulfurase gene *nifS* from *Azotobacter vinelandii* suggested that the *mal* gene encodes the enzyme that catalyzes sulfuration of Moco used by Xdh and Aox (Amrani et al., 2000; Ichida et al., 2001). Oxo-containing Moco does not function in Xdh and Aox, but is the cofactor for Suox (Figure 1).

## Fe-S and moco enzymes

The fly offers an ideal system for interdisciplinary research to bridge findings from genetic manipulations and resulting phenotypes with detailed biochemical studies to build functional understanding for animal cell physiology, keeping in mind the diversity of cell types and subcellular microenvironments. In this section, we review the enzymes that use Moco and/or Fe-S clusters. A representative list of these enzymes in *D. melanogaster* is provided (Table 1). We emphasize the physiological functions and biochemical pathways, as opposed to the detailed biochemistry of the cofactors at the active sites, which only in few cases has been the primary interest of *Drosophila* researchers.

**Table 1:** List of known molybdoenzymes, iron sulfur enzymes and scaffold proteins.

**Protein**	**Metal Cofactors**	**Cellular Compartment**	**Function**	**Key References**
**MOLYBDO-ENZYMES**				
**Aox 1-4** (aldehyde oxidases)	Moco 2 × [2Fe-2S]	Cytosol	Hydroxylation of xenobiotics	Dickinson and Gaughan, 1981; Marelja et al., 2014
**mArc** (mitochondrial amidoxime reducing component; CG1665)	Moco	Unknown	Unknown	Llamas et al., 2017
**Rosy** (xanthine dehydrogenase)	Moco 2 × [2Fe-2S]	Cytosol Pigment granule	Purine degradation Eye pigment formation	Keith et al., 1987; Reaume et al., 1991; Hilliker et al., 1992; Hughes et al., 1992; Kamleh et al., 2008
**Suox** (sulfite oxidase; CG7280)	Moco Heme	Mitochondrial intermembranes	H_2_S detoxification	Bogaart and Bernini, 1981; Braaten and Bentley, 1993
**MITOCHONDRIAL IRON SULFUR ENZYMES AND SCAFFOLD PROTEINS**				
**Acon** (mitochondrial aconitase)	[4Fe-4S]	Mitoch. matrix	Krebs cycle	Cheng et al., 2013; Esposito et al., 2013
**BolA 1-2**	[4Fe-4S]	Mitoch. matrix	2nd Fe-S assembly scaffold	Uzarska et al., 2016
**Fdx 1-2** (ferredoxins)	[2Fe-2S]	Mitoch. matrix	Electron transfer	Palandri et al., 2015
**Fech** (ferrochelatase)	[2Fe-2S]	Inner membrane	Heme biosynthesis	Sellers et al., 1998
**IscA 1-2** (magnetoreceptor?)	[4Fe-4S]	Mitoch. matrix	2nd Fe-S assembly scaffold	Mandilaras and Missirlis, 2012; Qin et al., 2016
**IscU**	[2Fe-2S] or [4Fe-4S]	Mitoch. matrix	1st Fe-S assembly scaffold	Dzul et al., 2017
**Las** (lipoic acid synthase)	2 × [4Fe-4S]	Inner membrane	Lipoate cofactor biosynthesis	Harmer et al., 2014
**Mocs1** (molybdenum cofactor synthesis 1)	[4Fe-4S]	Mitoch. matrix	Moco biosynthesis	Duke et al., 1975; Gray and Nicholls, 2000
**mtDNA-helicase**	[2Fe-2S]	Mitoch. matrix	Mitoch. DNA replication	Stiban et al., 2014
**ND-24** (NADH dehydrogenase) **ND-51** **ND-75** **ND-23** **ND-20**	[2Fe-2S] [4Fe-4S] [2Fe-2S] & 2 x [4Fe-4S] 2 x [4Fe-4S] [4Fe-4S]	Facing matrix Inner membrane	Oxidative phosphorylation Respiratory complex I	Zhang K. et al., 2013; Fiedorczuk et al., 2016; Garcia et al., 2017
**NFU 1-3**	[4Fe-4S]	Mitoch. matrix	2nd Fe-S assembly scaffold	Tong et al., 2003
**Nvd** (cholesterol 7 desaturase)	[2Fe-2S]	Mitoch. matrix	Steroid biosynthesis	Yoshiyama et al., 2006
**RFeSP** (Rieske iron-sulfur protein)	[2Fe-2S]	Inner membrane	Respiratory complex III	Gontijo et al., 2011
**SdhB** (succinate dehydrogenase)	[4Fe-4S]	Inner membrane	Respiratory complex II Krebs cycle	Au and Scheffler, 1994; Kohler et al., 1995; Gray et al., 1996; Walker et al., 2006; Na et al., 2014; Van Vranken et al., 2014
**CYTOSOLIC IRON SULFUR ENZYMES**				
**Cisd2** (CG1458) mitoNEET	[2Fe-2S]	Outer membrane	Fe-S cluster repair	Jones et al., 2014
**Elp3** (lysine acetyltransferase) Elongator complex protein 3	[4Fe-4S]	Cytosol Nucleus	Acetylates synapse active zone RNA transcript elongation	Miśkiewicz et al., 2011
**IRP 1A-1B** (cytosolic aconitases/ Iron regulatory proteins)	[4Fe-4S]	Cytosol	Cellular iron homeostasis Intermediary metabolism	Muckenthaler et al., 1998; Lind et al., 2006; Surdej et al., 2008
**Pixie** (RNAase L inhibitor, ABCE1)	2 × [2Fe-2S]	Cytosol	Ribosome biogenesis Translation initiation	Andersen and Leevers, 2007; Kashima et al., 2014
**Prat 1-2**	[4Fe-4S]	Cytosol	Purine nucleotide biosynthesis	Clark, 1994; Merzetti et al., 2013
**Su(r)** (CG2194) dihydropyrimidine dehydrogenase	[4Fe-4S]	Cytosol	Pyrimidine degradation	Van Gelder et al., 1995; Rawls, 2006
**NUCLEAR IRON SULFUR ENZYMES**				
**Chl1** (CG11403) DNA helicase	[4Fe-4S]	Nucleus	Heterochromatin organization	–
**DNApol-α180** (DNA polymerase) **DNApol-α60** (DNA primase) **DNApol-δ** (DNA polymerase)y**DNApol-ε255** (DNA polymerase) **DNA2** (CG2990) DNA helicase	[4Fe-4S] [4Fe-4S] [4Fe-4S] [4Fe-4S] [4Fe-4S]	Nucleus	Nuclear DNA replication	Kaguni et al., 1983; Peck et al., 1992; Sahashi et al., 2014
**Nthl1** (CG9272) DNA glycosylase	[4Fe-4S]	Nucleus	Base excision repair	–
**Xpd** (xeroderma pigmentosum D)	[4Fe-4S]	Nucleus	Subunit of basal transcription and DNA repair factor TFIIH	Reynaud et al., 1999; Chen et al., 2003; Aguilar-Fuentes et al., 2006; Li et al., 2010

### The molybdoenzymes: Xdh, Aox, Suox

In *D. melanogaster*, the *rosy* gene encodes Xdh (Chovnick et al., 1976; Keith et al., 1987). *Rosy* mutants accumulate xanthine and hypoxanthine and are devoid of urate (Hadorn and Schwinck, 1956; Morita, 1958; Glassman and Mitchell, 1959) and show a dull reddish-brown eye color because of the lowered concentrations of the red eye pigment drosopterin (bright red), and the increased chromogenic oxidation of the eye pigment dihydroxanthommatin (yellow brown) to xanthommatin (dull dark brown), either due to enzymatic activity (Phillips and Forrest, 1980; Wiley and Forrest, 1981; Ferre et al., 1986) or due to the decreased urate concentration (Hilliker et al., 1992). The use of *rosy* mutant strains to probe the structure and function of Xdh provides an early, elegant example in the field of biological inorganic chemistry (Hughes, 1992; Hughes et al., 1992; Doyle et al., 1996). Allelic series of mutants affecting residues coordinating the Fe-S clusters, the Moco, the flavin cofactor or the binding sites for NAD^+^/NADH were used to decipher the complex mechanism of action for this prototypical molybdoenzyme. More recently, metabolic profiling of *rosy* mutants revealed additional changes in the tryptophan, arginine, pyrimidine, and glycerophospholipid metabolic pathways (Kamleh et al., 2008, 2009).

Two genes, *low pyridoxal oxidase* (*lpo*) and *aldehyde oxidase* (*Aldox*), were initially recognized to encode Aoxs in *D. melanogaster* (Courtright, 1967; Collins and Glassman, 1969; Dickinson, 1970; Browder and Williamson, 1976; Dickinson and Gaughan, 1981; Cypher et al., 1982; Nelson and Szauter, 1992). Genome analysis revealed, however, a cluster of four Aox genes (Garattini et al., 2008). We showed previously that (i) the *lpo* gene encodes Aox1, (ii) Aox2 activity is only present during metamorphosis, (iii) the activities associated with the classic *Aldox* locus correspond to two splice forms of the *Aox3* gene, and (iv) no major activity has been found associated with Aox4, the newest addition to a highly evolving protein family in Drosophilidae (Marelja et al., 2014). Aoxs show specific substrate specificities, but their *in vivo* substrates and physiological functions remain unclear (Dickinson and Gaughan, 1981; Cypher et al., 1982; Marelja et al., 2014).

Suox contains a cytochrome b5 (heme-containing) domain followed by 12–15 amino acids connecting it to the Moco domain. Suox catalyzes an oxygen atom transfer reaction to sulfite leading to its oxidation (and detoxification) to sulfate (Feng et al., 2007; Hille et al., 2011), an activity previously detected in *Drosophila* (Bogaart and Bernini, 1981; Braaten and Bentley, 1993). Based on sequence homology, we find that *CG7280* encodes for the *Drosophila* Suox.

A novel molybdoenzyme, mitochondrial amidoxime reducing component (mArc), has been described and has an identifiable *Drosophila* homolog, CG1665. The enzyme from humans has been proposed to reduce a broad range of N-hydroxylated compounds receiving electrons from cytochrome b5 (Gruenewald et al., 2008) and to reduce nitrite to nitric oxide (Sparacino-Watkins et al., 2014). A number of controversies over the function, subcellular localization and whether the newly discovered enzyme binds to sulfurated or oxo Moco, have been reviewed (Llamas et al., 2017).

Another relatively unexplored area in *Drosophila* molybdenum biology relates to the trafficking and homeostatic mechanisms for handling dietary molybdate. In wild type flies, Xdh and Aox activities are relatively stable, not responding to concentrations between 1 and 10 mM molybdate (Duke et al., 1975), although more recent studies documented a number of, so far unexplained, sex-specific physiological responses in carbohydrate and thiol metabolism at the same concentrations (Rovenko et al., 2014; Perkhulyn et al., 2017). Interestingly, 50 mM molybdate was tolerated by wild type flies, but was lethal to *Mocs1* mutant flies, implicating the Moco biosynthetic pathway as part of the detoxification mechanisms available to the fly (Duke et al., 1975).

### The curious case of the *Maroon* mutant

A second unusual aspect of Xdh, besides its implication in multiple metabolic pathways (see above Kamleh et al., 2008, 2009), relates to the enzyme's trafficking to the eye imaginal disk, where it accumulates in pigment granules (Reaume et al., 1989, 1991). Due to the similar eye color of the classic *maroon* mutant with that of the *rosy* and *mal* mutants (Bridges, 1918), we assayed *maroon* fly extracts for Xdh and Suox activity, expecting to find decreased Xdh activity consistent with the eye phenotype. To our surprise, increased activities of both enzymes compared to wild type flies were observed in *maroon* extracts (Figure 7). As Suox utilizes unmodified Moco, the increase in its activity was not entirely unexpected, because this form of Moco might accumulate as in *rosy* and *mal*. Accounting for increased Xdh activity was harder, however, given the similar eye color between *maroon, rosy*, and *mal*. The demonstration that the *maroon* gene encodes for Vacuolar protein sorting 16A (Vps16A; Grant et al., 2016), a protein implicated in granule formation (Pulipparacharuvil et al., 2005; Lorincz et al., 2016), suggests a possible defect in the tissue localization of Xdh in *maroon* mutants, as the enzyme obviously remains functional in whole fly extracts and is even induced (Figure 7). It will be informative to test in *maroon* mutants whether the Xdh activity is localized in peripheral tissues, such as the Malpighian tubules, and not in the eyes. If this prediction is correct, then *Vps16A* may represent the first known mutant that blocks the delivery of a vesicular structure to the eye. Another unresolved piece of this puzzle relates to the way in which Xdh, a cytosolic enzyme, is found in pigment granules in the eye (Reaume et al., 1989, 1991). The discovery of exosomes gives a possible answer to this conundrum (Hemler, 2003; Gross et al., 2012; Gradilla et al., 2014; Takeuchi et al., 2015; Beer and Wehman, 2017; Shibata et al., 2017; Tassetto et al., 2017). Clearly more experiments are required to explain how Xdh acts in the formation of eye color in flies, but complex non-cell autonomous processes relating to enzyme maturation, regulation, and transport are involved.

### Mitochondrial Fe-S proteins at the heart of mitochondrial bioenergetics

Mitochondria form an important organelle of eukaryotic cells, typically containing their proper genome (Lane and Martin, 2010; Schatz, 2013; Allen, 2015) and performing various functions (Pagliarini and Rutter, 2013; Chandel, 2015; Munro and Treberg, 2017), of which the tricarboxylic acid (TCA) cycle and oxidative phosphorylation are famous (Vakifahmetoglu-Norberg et al., 2017). The idea that iron plays a part in the oxidation reactions of the living cell was firmly established by Warburg (1925). Beinert and Sands interpreted electron paramagnetic resonance spectra of succinate dehydrogenase (Sdh) as “non heme iron” (Beinert and Sands, 1960; Beinert, 2002). Later, Sdh and aconitase (both TCA cycle enzymes) were shown to carry [4Fe-4S] clusters (Ruzicka and Beinert, 1978; Cammack, 1982). The *Drosophila* enzymes are no exception to the rule (Duke, 1968; Fox et al., 1972; Au and Scheffler, 1994; Vincent et al., 2012; Esposito et al., 2013). Failure to build these Fe-S clusters will inevitably block the TCA cycle, and thereby development and growth (Au and Scheffler, 1994; Yan et al., 1997; Walker et al., 2006; Uhrigshardt et al., 2013; Na et al., 2014). Sdh is also known as Complex II of the respiratory chain that generates the inner mitochondrial membrane potential and proton gradient used by the F-ATPase for the production of ATP during oxidative phosphorylation (Alziari et al., 1985; Sardiello et al., 2005; Liu et al., 2011; Barry and Thummel, 2016). Complex I of the respiratory chain, otherwise known as NADH dehydrogenase, carries eight precisely spaced Fe-S clusters of different reduction-oxidation potentials assembled on five subunits of the complex (Table 1; Zhang K. et al., 2013; Fiedorczuk et al., 2016; Garcia et al., 2017). It is thus plain that without Fe-S clusters ATP cannot be produced in the mitochondrial process of aerobic respiration (Anderson et al., 2005; Llorens et al., 2007; Navarro et al., 2011; Edenharter et al., 2017).

### Fe-S enzymes are required for heme, ecdysone, and lipoate biosynthesis

Ferrochelatase is an enzyme that resides in the inner mitochondrial membrane accepting iron from the mitochondrial matrix and protoporphyrin IX from the intermembrane space to generate heme (Wu et al., 2001). Heme is another abundant iron-dependent protein cofactor (Ponka et al., 2017). Many ferrochelatases, including the *Drosophila* enzyme, carry a [2Fe-2S] cluster (Sellers et al., 1998). Therefore, both major forms of iron cofactors used in biology rest on the mitochondrial Fe-S cluster assembly machinery.

Furthermore, *Drosophila* ferredoxins carry a [2Fe-2S] cluster required for electron transfer during Fe-S cluster assembly, but also for the production of ecdysone in the larval prothoracic gland and other steroidogenic tissues (Uhrigshardt et al., 2013; Palandri et al., 2015). In this way, iron availability is linked to a key developmental signal that terminates growth and initiates metamorphosis (Yamanaka et al., 2013; Sandoval et al., 2014). Interestingly, glutathione production in the prothoracic gland is also required for steroidogenesis (Enya et al., 2017). The possibility that glutathione supports Fe-S cluster biosynthesis in this tissue should be considered (Song et al., 2006; Auchère et al., 2008; Qi et al., 2013; Ozer et al., 2015).

Lipoic acid or lipoate is a cofactor required in intermediary metabolism enzymes α-oxoglutarate dehydrogenase, pyruvate dehydrogenase, branched-chain oxoacid dehydrogenase, 2-oxoadipate dehydrogenase, and in the glycine cleavage system (Habarou et al., 2017). Biosynthesis of the lipoate cofactor is not well understood beyond prokaryotes (for comprehensive review see Cronan, 2016). Nevertheless, it is clear that lipoic acid synthase is required for the maturation of enzymes dependent on lipoate and uses two [4Fe-4S] clusters for its catalytic activity (Harmer et al., 2014). No study describing the *Drosophila* lipoic acid synthase has been published despite human disease related to lipoic acid deficiency (Mayr et al., 2014; Cronan, 2016; Habarou et al., 2017).

### The central dogma of molecular biology depends on Fe-S enzymes

The central dogma of molecular biology, originally proposed by Crick (1958), radically changed the way biology is understood and taught (Cobb, 2017). DNA replication is the primary mode of information transfer during successive generations, whereas DNA transcription is the primary mechanism for specifying which proteins can be translated on ribosomes assembled in the cytoplasm. In addition to the nucleotide and amino acid building blocks, all three steps require energy and are interdependent, as nucleic acids and proteins are both essential for each process. Likewise, Fe-S clusters are also required at each step as cofactors of the DNA polymerase (see references in Table 1 and Kaguni et al., 1983; Peck et al., 1992; Sahashi et al., 2014; Stiban et al., 2014), of the essential subunit of the basal transcription factor TFIIH Xpd (Reynaud et al., 1999; Chen et al., 2003; Aguilar-Fuentes et al., 2006; Li et al., 2010) and of Pixie, which is required for ribosome biosynthesis and the initiation of translation (Andersen and Leevers, 2007; Kashima et al., 2014). Thus, DNA replication, transcription, and translation rest on the CIA providing Fe-S cluster to DNA polymerase, Xpd, and Pixie, respectively.

### Are Fe-S and/or moco enzymes implicated in the circadian clock?

Plants alternate between photosynthesis and respiration during day-night cycles, whereas animal behavior shifts between an active stage that includes foraging, feeding and other motile behaviors, and sleep (Haydon et al., 2013; Mellor, 2016; Dubowy and Sehgal, 2017). Most animals anticipate the periodicity of sunlight and darkness through dedicated neuronal circuits whose rhythmic activity is sometimes referred to as the circadian clock (the genetic basis of which, was first discovered by Konopka and Benzer, 1971). In *Drosophila* the organization of the circadian circuitry has received considerable attention (Nitabach and Taghert, 2008; Hermann et al., 2013; Simoni et al., 2014). Similar to other animals, the circadian clock is interlinked with physiological functions in flies (Barber et al., 2016; Katewa et al., 2016; Rey et al., 2016; Kijak and Pyza, 2017; Klemz et al., 2017). Given the major role of Fe-S enzymes in intermediary metabolism and aerobic respiration, the question of whether Fe-S clusters are continuously present in key enzymes during the day-night cycle or whether some recycling of iron takes place in a rhythmic function has been posed (Mandilaras, 2012).

**Figure 8:**
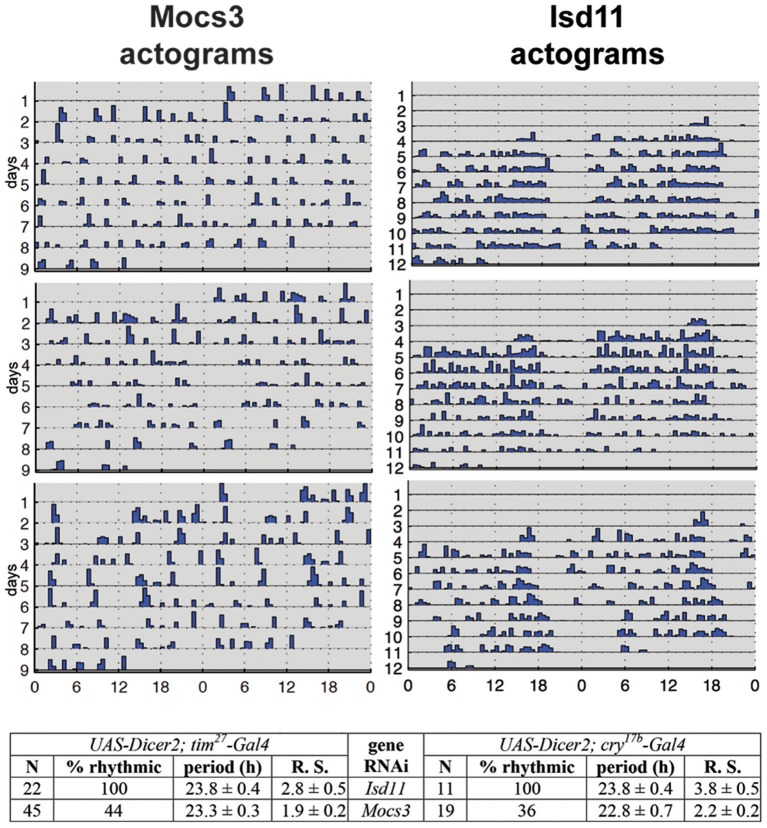
RNAi of *Mocs3* disrupts circadian activity in constant darkness. Flies were first entrained on 3 12:12 h light-dark cycles then representative actograms are presented during constant darkness. Statistical analysis is depicted for two independent drivers. N represents number of individuals tested, Rhythmic Statistic (R. S.) values are provided for flies scored as rhythmic: higher values demonstrate more robust rhythmicity (for detailed methodology see Mandilaras and Missirlis, 2012).

RNAi of *Nfs1* (the cysteine desulfurase required for the biosynthesis of Fe-S clusters and Moco; Figure 1) in the circadian clock neurons resulted in loss of rhythmic activity of flies monitored under constant darkness (Mandilaras and Missirlis, 2012). Ubiquitous RNAi of *Nfs1* caused lethality and eye-specific RNAi caused photoreceptor cell loss (Marelja, 2013). Two driver lines with overlapping, but not identical, expression patterns in the clock neurons, *tim*^27^*-Gal4* and *cry*^17*b*^*-Gal4* were recombined to the RNAi potentiator *UAS-Dicer2* (Dietzl et al., 2007) and used, showing that *Nfs1* RNAi driven by *tim*^27^*-Gal4* resulted in a weaker arrhythmia than when driven by *cry*^17*b*^*-Gal4* (Mandilaras and Missirlis, 2012). *IscU* RNAi driven by *cry*^17*b*^*-Gal4* also resulted in arrhythmic flies, *IscU* RNAi driven by *tim*^27^*-Gal4*, however, resulted in lethality (a more severe phenotype, but one that cannot be tested for rhythmicity). In contrast, *frataxin* RNAi with both drivers did not show an arrhythmic phenotype (Mandilaras and Missirlis, 2012). To probe these genetic results further, RNAi of *Isd11* and *Mocs3* using the same drivers and assay was undertaken. *Isd11* RNAi in circadian clock neurons showed no discernible phenotype, in contrast to *Mocs3* RNAi, which resulted in 56% arrhythmic flies when driven with *tim*^27^*-Gal4* and 64% arrhythmic flies when driven with *cry*^17*b*^*-Gal4* (Figure 8).

The other Fe-S cluster biosynthesis genes that were implicated in the *Drosophila* circadian clock were *Iba57* (*CG8043*) and *Cfd1* (*CG4858*; also referred to as *Nubp2*), both encoding components of the CIA machinery, and *IscA1*, which is an Fe-S scaffold protein predicted to be localized to mitochondria (Mandilaras and Missirlis, 2012). *IscA1* has recently found an exciting application in a new, non-invasive, technology to control experimentally the generation of neuronal action potentials, termed magnetogenetics (Long et al., 2015). It was also suggested that *IscA1* is a component of fly magnetoreception (Cyranoski, 2015; Qin et al., 2016), but see (Meister, 2016; Pang et al., 2017). The proposed cryptochrome-IscA1 protein complex is predicted to reside in the cytosol or nucleus, given that cryptochrome is a protein localized in the cytosol and nucleus (Stanewsky et al., 1998; Yoshii et al., 2008). Whether, in addition to their predominant presence in mitochondria, IscA1, IscU, and Nfs1 have cytosolic localization in flies, as recently shown for human HSC20 (Kim et al., 2018), requires experimental verification. It is an interesting possibility to keep in mind when considering the result that RNAi against *Mocs3*, whose product is the proposed cytosolic partner of Nfs1 (Marelja et al., 2008, 2013), resulted in arrhythmic flies (Figure 8). Of note, two Fe-S enzyme encoding genes, dihydropyrimidine dehydrogenase and phosphoribosylamidotransferase, show circadian expression and are localized in the cytosol (Van Gelder et al., 1995; Rey et al., in review).

The new results presented here and the findings in Mandilaras and Missirlis (2012) are only based on RNAi and require independent confirmation. That iron may play a role in the circadian clock and sleep disorders was also proposed based on a *Drosophila* model of the human Restless Legs Syndrome (Freeman et al., 2012, 2013). This line of research was unfortunately disrupted at Queen Mary University of London (Allen and Missirlis, 2012; Ashworth, 2012; Horton, 2012), but deserves further attention, given the interplay between iron metabolism and the circadian clock in humans (Earley et al., 2014; Furudate et al., 2014; Dye et al., 2016), pigs (Zhang et al., 2017), rodents (Yin et al., 2007; Bianco et al., 2009; Simcox et al., 2015; Janich et al., 2016; Okazaki et al., 2016), plants (Chen et al., 2013; Hong et al., 2013; Salomé et al., 2013), and even diatoms (Botebol et al., 2015).

### Cellular iron sensing and regulation is coupled to Fe-S cluster biosynthesis

*D. melanogaster* larvae or flies grown on diets with different iron content show cell-type specific responses (Poulson and Bowen, 1952; Georgieva et al., 1999; Mehta et al., 2009; Mandilaras et al., 2013). In mammals, IRPs regulate cytosolic iron concentrations (Zhang et al., 2014; Muckenthaler et al., 2017; Papanikolaou and Pantopoulos, 2017; Rouault and Maio, 2017). *D. melanogaster* has two IRP-like proteins, showing a partial evolutionary conservation in its iron sensing mechanism (Muckenthaler et al., 1998; Lind et al., 2006; Freeman et al., 2013). Another conserved aspect in cellular iron homeostasis is that genetic manipulations that affect mitochondrial Fe-S cluster biosynthesis lead to cytosolic iron depletion (Anderson et al., 2005; Uhrigshardt et al., 2013; Navarro et al., 2015). The mechanism of this response is not fully conserved between yeast (Wofford and Lindahl, 2015) and vertebrates (Wingert et al., 2005; Ye et al., 2010), as the former lack IRPs, and it is not known how *Drosophila* cytosolic iron is regulated through the mitochondrial Fe-S cluster assembly machinery. Furthermore, transcriptional regulation of ferritin upon iron sensing appears to dominate the *Drosophila* iron response (Georgieva et al., 1999; Missirlis et al., 2007; Rosas-Arellano et al., 2016), suggesting that an undiscovered transcription factor responds to varying cytosolic iron concentration in insects. Of note, *Drosophila* ferritin is loaded with iron in the endoplasmic reticulum (Xiao et al., 2014; Xiao and Zhou, 2018) and is an essential gene (González-Morales et al., 2015), as is also the case for IRP-1A (Puri et al., 2008). More work is needed to understand cellular iron homeostasis in *Drosophila*.

### How do mitochondria regulate their iron needs?

**Figure 9:**
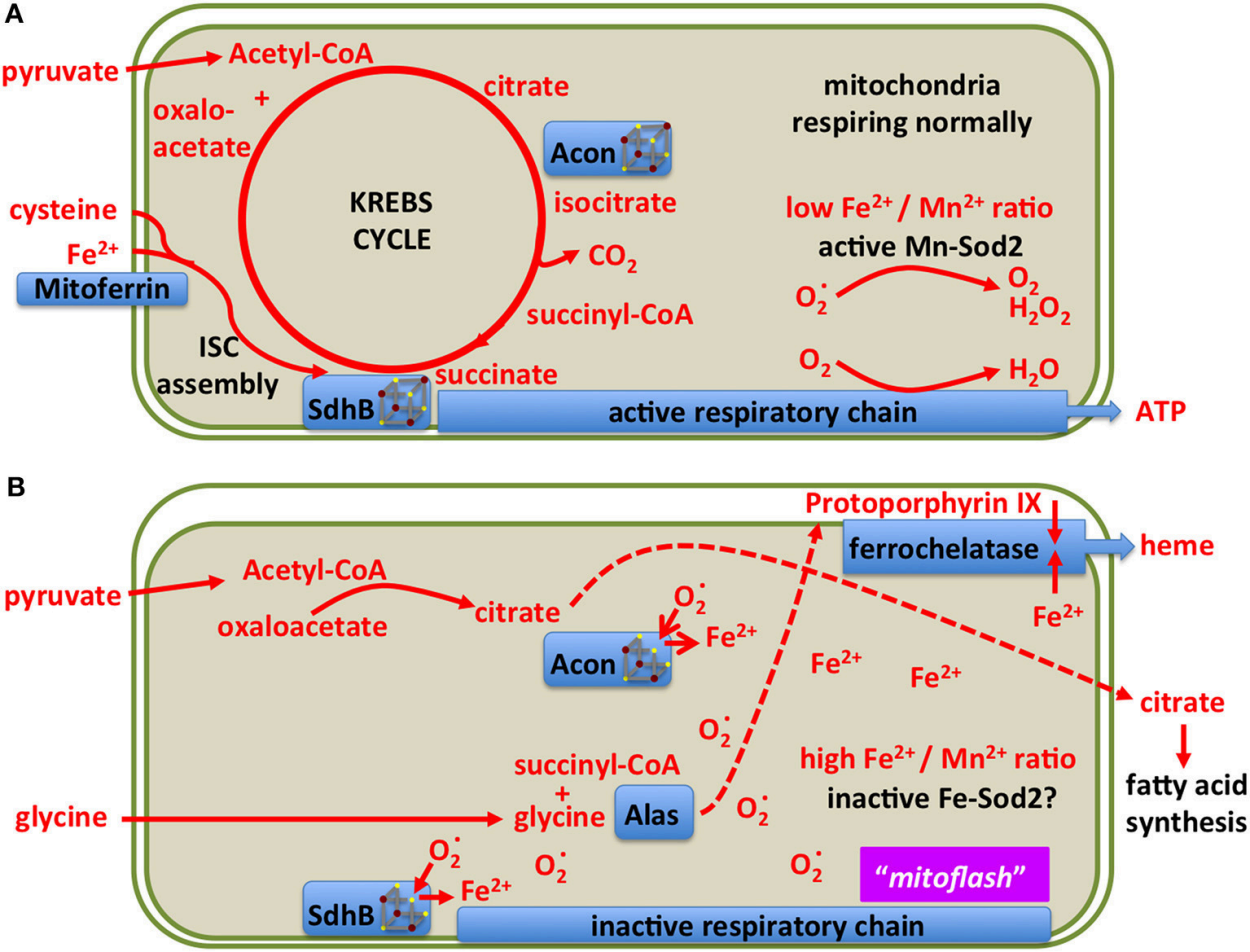
A model showing two different states of mitochondrial metabolism. **(A)** Conventional state based on the TCA (Krebs) cycle feeding reducing equivalents into the respiratory chain for the production of ATP. Only metabolites and enzymes referred to in the text are shown for simplicity. **(B)** A superoxide burst is predicted to reduce the [4Fe-4S] clusters of aconitase and Sdh, releasing ferrous iron into the mitochondrial matrix and blocking the TCA cycle. The resulting increase in iron concentration may inactivate manganese Sod2 resulting in a sustained pulse of superoxide, previously referred to as a “mitoflash.” In this state, mitochondria export citrate to the cytosol for fatty acid production, Alas uses up succinyl-CoA for protoporphyrin biosynthesis, ferrochelatase uses protoporphyrin and iron to produce heme. The consequent drop in matrix soluble iron reactivates manganese Sod2, allowing for Fe-S cluster biosynthesis to reactivate the TCA cycle enzymes.

Mitochondria use a specialized ferritin (Missirlis et al., 2006) and at least one specialized transporter for iron import into the mitochondrial matrix, mitoferrin (Metzendorf and Lind, 2010; Navarro et al., 2015; Edenharter et al., 2017). The question how they sense and regulate iron concentration in the matrix is not resolved. One unexplained observation in this respect is that, at least in yeast, the GTP to GDP ratio affects iron concentration within this compartment (Gordon et al., 2006). In what follows, we explore the idea that manganese Sod2 (Kirby et al., 2002; Duttaroy et al., 2003) may serve as a mitochondrial iron sensor (Figure 9). Our view of normally respiring mitochondria is the familiar setting with an active Sod2 protecting Fe-S clusters and maintaining the TCA cycle (Figure 9A; Missirlis et al., 2003a). An increase in superoxide (Wong H. S. et al., 2017) deactivates the Fe-S clusters in aconitase and Sdh (Gardner and Fridovich, 1991; Gardner et al., 1995) leading to an increase in ferrous iron in the mitochondrial matrix (Srinivasan et al., 2000; Jensen et al., 2004; Esposito et al., 2013). The literature on these reactions has been reviewed with a discussion of the accompanying consequences for cell metabolism (see superoxide/aconitase rheostat model; Armstrong et al., 2004). Increased mitochondrial iron can replace manganese on Sod2 and inactivate the enzyme (Yang et al., 2006; Naranuntarat et al., 2009). Sod2 inactivation would result in a positive feedback loop, as more superoxide would accumulate, fully inactivating aconitase and Sdh bringing the TCA cycle to a halt.

Heping Cheng and co-workers described a superoxide burst in mitochondria, termed mitoflash, lasting for several seconds (Wang et al., 2008; Shen et al., 2014). Some skepticism on whether the phenomenon is real exists, mostly arguing that perhaps the change in the fluorescence of the reporter used relates to pH changes (Schwarzländer et al., 2012, 2014), but the coupling of TCA cycle with oxidative phosphorylation could mean that both claims (a burst in superoxide and alkalinization of the mitochondrial matrix) occur at similar timeframes or in parallel (Wei-LaPierre et al., 2013).

During the inactivation of Sod2, citrate cannot be isomerized to isocitrate and is therefore exported to the cytosol for fatty acid synthesis (see below). Likewise succinate cannot be metabolized, but its precursor, succinyl-CoA is a substrate of aminolevulinate synthase (Alas) in the first step of heme biosynthesis (Ruiz de Mena et al., 1999; Ponka et al., 2017). Thus the superoxide burst couples the redirection of iron and intermediary metabolites to ferrochelatase for heme production (Figure 9B). This way, iron concentration in the mitochondrial matrix drops, manganese binds to and reactivates Sod2, and the mitochondria return to their conventional state. In the above discussion we have not considered the role of hydrogen peroxide, produced by Sod2, which should be treated as a distinct metabolite (Missirlis et al., 2003b; Munro and Treberg, 2017).

Although the metabolites that are affected in the *Sod2* mutants remain to be described, a recent paper reported a four-fold induction of aminolevulinic acid in *Sod1* mutants, and curiously, a 20-fold induction of citrulline (Doran et al., 2017). Citrulline production depends on (the heme-containing) nitric oxide synthase (Nos), which uses as substrate arginine (Kuzin et al., 1996; Jaszczak et al., 2015). Superoxide-dependent inactivation of another manganese-containing enzyme, arginase, which degrades arginine (Samson, 2000), could lead to increased substrate availability for Nos. Indeed, it has been shown that arginase inactivation induces Nos activity (Caldwell et al., 2015). The above suggest another testable example of a similar hypothesis, where the interaction of superoxide with Fe-S clusters influences cell metabolism by releasing iron, which could transiently displace manganese from the active site of its target enzyme(s).

### Fe-S clusters and lipid metabolism

Citrate is a key precursor for fatty acid synthesis (Watson and Lowenstein, 1970; Halperin et al., 1975) and therefore it is not surprising that inactivation of aconitase leads to increased lipogenesis (reviewed in Tong and Rouault, 2007). The same metabolic connection has also been demonstrated in *Drosophila*, by driving *frataxin* RNAi in glial cells (Navarro et al., 2010) or in photoreceptor mutant clones (Chen et al., 2016b). Another outcome of increased mitochondrial iron due to loss of *frataxin* is the activation, in a way that is not yet understood, of sphingolipids. Sphingolipids, in turn, activate signal transduction pathways, like, in the example of photoreceptors, of Phosphoinositide-dependent kinase 1 and Myocyte enhancer factor 2 (Chen et al., 2016a,b). Moreover, murine mutants in Sod2 show increased lipid accumulation in their livers (Li et al., 1995; Chouchani et al., 2017), which we attribute to the inactivation of aconitase (Figure 9). It remains to be shown whether during normal physiology our hypothesis of two alternating mitochondrial states, one driving aerobic respiration, the other driving lipogenesis and heme biosynthesis, is valid or not.

## Physiological relevance of Fe-S and moco enzymes in different tissues

In this section we first look at the role of Fe-S enzymes during the life cycle of *D. melanogaster*, then focus on the main tissues of the adult fly where functions of Fe-S and Moco enzymes are known.

### Development, growth, and aging: stem cells and cellular differentiation

The life cycle of *Drosophila* is separated in distinct stages. Egg-laying follows the insemination of oocytes in the female, embryogenesis gives rise to the larva, which feeds and grows until entry into metamorphosis, at the end of which adult flies emerge (Demerec, 1950). We discussed above that Fe-S enzymes drive aerobic respiration, which changes dramatically with the progress of embryogenesis (Lints et al., 1967; Tennessen et al., 2011, 2014), during larval growth (Heinrich et al., 2011; Merkey et al., 2011; Sen et al., 2013; Da-Ré et al., 2014) and adult aging (Lints and Lints, 1968; Ferguson et al., 2005; Dubessay et al., 2007; Klichko et al., 2014; Wolff et al., 2016). The decline in mitochondrial functions observed in late life, along with experiments showing that genetic manipulations leading to improved mitochondrial functions extended lifespan, suggested that mitochondrial metabolism governs the adult life span (Villee, 1948; Miquel, 1998; Fukagawa, 1999; Ross, 2000; Muller et al., 2007), but the finding that mitochondria isolated from old flies incubated with cytosol from young individuals restore respiration, whereas mitochondria from young individuals incubated with cytosol of old individuals fail to produce ATP questions this view (Vann and Webster, 1977; see also Sanz, 2016). On the other hand, there can be no doubt that defects in Fe-S cluster biosynthesis inhibit growth and dramatically shorten lifespan (Missirlis, 2003). It is also good to remember that the two sexes need to be considered separately when studying mitochondrial metabolism (Camus et al., 2012, 2015; Pomatto et al., 2017).

Increased glycolysis uncoupled from aerobic respiration is a characteristic of proliferative cells, for example during the early stages of *Drosophila* embryogenesis (Tennessen et al., 2011, 2014). This major metabolic switch is mediated through cell signaling (Thörig et al., 1981a,b; Markopoulou and Artavanis-Tsakonas, 1989; Homem et al., 2014; Barry and Thummel, 2016; Sieber et al., 2016; Slaninova et al., 2016; Mattila and Hietakangas, 2017). We already discussed that exit from the growth stage requires the concerted activity of Fe-S and heme enzymes in the prothoracic gland for ecdysone synthesis (Llorens et al., 2015; Palandri et al., 2015). Further, ecdysone is one of the signals inducing oxidative phosphorylation through the mitochondrial respiratory chain, sensed by neuroblasts and leading to their terminal differentiation (Homem et al., 2014) (see also Sen et al., 2013). Iron itself can directly influence stem cell differentiation, exemplified by hemocyte production in the lymph gland (Yoon et al., 2017). Thus, primary cell metabolism can define the fate of stem cells in a developing organism.

### The germ line: is a lineage of quiescent mitochondria set aside for reproduction?

**Figure 10:**
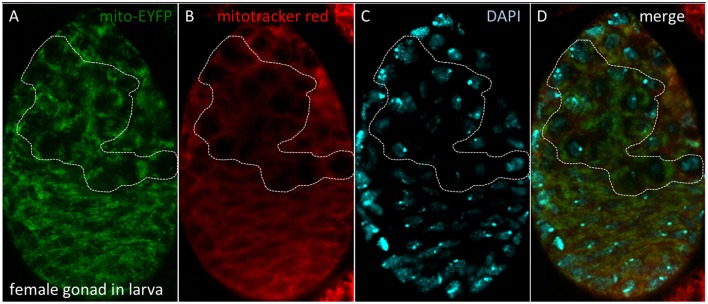
Quiescent mitochondria in female gonads of *D. melanogaster* larvae. **(A)** Bloomington stock #7194 carries a P-element insertion including the *spaghetti squash* promoter driving expression of EYFP tagged at the N-terminal end with a mitochondrial targeting sequence. **(B)** Dissected gonads were incubated with Mitotracker Red, which accumulates in mitochondria with active membrane potential. **(C)** Nuclei were observed with DAPI. **(D)** Merged image. The dotted line depicts the female germ line, where quiescent mitochondria are observed. Methods as described in Cox and Spradling (2003).

Stem cell differentiation in the ovary was found to depend on the mitochondrial ATP synthase (Teixeira et al., 2015). Surprisingly, in the female germarium, oxidative phosphorylation played no role in the early differentiation steps of the female germ line (Teixeira et al., 2015). Why are differentiating stem cells in the ovary and the larval brain different? One idea, first proposed by Allen (1996), is that the female germ line is defined as a carrier of quiescent (non-respiring) mitochondria (Cox and Spradling, 2003; de Paula et al., 2013a,b; Sieber et al., 2016). A key experiment to test this hypothesis is whether quiescent mitochondria can be detected throughout the life cycle in the female germ line (Allen and de Paula, 2013). We showed that quiescent mitochondria are observed in the female germ line within gonads of third instar larvae (Figure 10). However, similar experiments have proven harder to perform in embryos due to impermeability of the Mitotracker Red and reduced *spaghetti squash*-EYFP fluorescence (LaJeunesse et al., 2004), although the laboratory of Richa Rikhy recently succeeded to image mitochondria in living embryos using new constructs (Chowdhary et al., 2017). Of note, these quiescent mitochondria would constitute a third mitochondrial state, not described in Figure 9, since they lack the oxidative phosphorylation complexes and hence a source of superoxide to protect the mitochondrial DNA. This line of research was also unfortunately disrupted at Queen Mary University of London (Allen and Missirlis, 2012; Ashworth, 2012; Horton, 2012); for further insights see (Burrows, 2012; Mahul-Mellier et al., 2015; Lawrence, 2016; Edwards and Roy, 2017; Tsimilli-Michael and Haldimann, 2017).

Alan Spradling and co-workers have also described quiescent mitochondria in the oocyte (Cox and Spradling, 2003), but they have shown in addition an active mitochondrial membrane potential during stages 4–8 of oogenesis (Sieber et al., 2016). Insulin signaling acting through glycogen synthase kinase 3 is required to set mitochondria into a quiescent phase from stage 10 of oogenesis (Sieber et al., 2016). Is the finding of an active membrane potential during oogenesis sufficient to refute the theory of quiescent mitochondria? The first issue to resolve is whether mitochondria in the germarium respire or are quiescent. Our interpretation of the limited published data (Sieber et al., 2016) is that mitochondria in female germ cells are quiescent and this would offer an explanation for the unexpected finding that the early steps of the female germ line differentiation is independent of oxidative phosphorylation (Teixeira et al., 2015). However, another well-known phenomenon in the transmission of mitochondria through successive generations is bottleneck selection of functional mitochondria, which occurs during *Drosophila* oogenesis (Cox and Spradling, 2003, 2006; Hill et al., 2014; Ma et al., 2014; Chen Z. et al., 2015). It is therefore possible that the discovery by Ruth Lehmann and co-workers of ATP synthase induction in the first steps of such a differentiation program proceeds in order for mitochondria to test their ability to sustain a membrane potential during stages 4–8 of oogenesis as a selection of functional mitochondria that can be safely transmitted to the oocyte.

If Allen's hypothesis is proven correct, he may have resolved Darwin's still-standing question of why it is that two separate sexes evolved (Venton, 2013). The two sexes were required to keep a quiescent form of mitochondria in the resting gamete, while dispensing the mitochondria used by the motile gamete (DeLuca and O'Farrell, 2012; Politi et al., 2014). The mitochondrial DNA polymerase (an Fe-S enzyme, Stiban et al., 2014) participates in the elimination of paternal mitochondrial genomes in *D. melanogaster* by a mechanism that is not yet understood (Yu et al., 2017).

### Eye pigment formation

The study of heredity defines the birth of the field of *Drosophila* research, famously through observations of the color of their eyes (Morgan, 1910, 1911). The study of how eye pigments are formed, inspired by the drive to understand the genetic control of development (Beadle and Ephrussi, 1936; Lewis, 1978), gave rise to biochemical genetics. Progress in biochemistry (i.e., Hadorn and Mitchell, 1951) made clear early on that the Moco is required for pigment biosynthesis (Glassman and Mitchell, 1959; Hubby and Forrest, 1960). Progress in electron microscopy revealed impressive changes in cell biology occurring in eye color mutants (Nolte, 1961; Shoup, 1966; Sullivan and Sullivan, 1975) and, as discussed above, how Xdh reaches the pigment cells of the eye remains unsolved (Reaume et al., 1989, 1991).

### Malpighian tubules and urate excretion

Malpighian tubules are the major excretory organ in flies (Beyenbach et al., 2010). The primary role of Xdh is in purine catabolism, mostly taking place in the Malpighian tubules (Dickinson and Gaughan, 1981; Reaume et al., 1989). Zinc ions are implicated in mineral excretion through this organ (Chi et al., 2015; Yin et al., 2017), while the same genes that govern pigment granule formation in the eye are also required for the formation of zinc storage granules in the Malpighian tubules (Tejeda-Guzman et al., 2018). Finally, the Malpighian tubules also show strong Aox activity (Dickinson and Gaughan, 1981), although the physiological function of this activity remains to be shown (Marelja et al., 2014).

### Muscles for flight and heartbeat

*D. melanogaster* is able to fly several kilometers in the open desert (Dickinson, 2014). To do so, it uses specialized muscles (Iwamoto, 2011), which receive oxygen directly through the trachea (Lehmann and Schützner, 2010), respiring over 90% of the oxygen to sustain flight (Suarez, 2000). Mitochondria are key to this action (Levenbook and Williams, 1956) and ambient oxygen concentrations alter flight performance (Skandalis et al., 2011; Bosco et al., 2015; Shiehzadegan et al., 2017). If Fe-S respiratory enzymes are affected either by aging (Ferguson et al., 2005) or by mutation (Walker et al., 2006; Godenschwege et al., 2009; Martin et al., 2009; Vrailas-Mortimer et al., 2011; Oka et al., 2015), muscle pathology ensues. On the other hand, flies are highly resistant to hypoxia and indeed lacking manganese Sod2 results in short-lived adults (the majority dying within the first couple of days from pupal eclosion; Godenschwege et al., 2009), but this mortality is rescued by moving the flies in a hypoxic environment (Wicks et al., 2009). A survey into the genetic factors regulating natural variation in mitochondrial function in the *Drosophila* muscle revealed nuclear genomic control of naturally occurring variation in mitochondrial respiration (Correa et al., 2012; Jumbo-Lucioni et al., 2012), a process also regulated by calcineurin (Pfluger et al., 2015) and the mitochondrial contact site and cristae junction organizing system (Guarani et al., 2015). Finally, the requirement of Fe-S clusters and the respiratory chain has also been demonstrated in the *Drosophila* heart muscle (Tricoire et al., 2014; Martínez-Morentin et al., 2015).

### The insect intestine functions beyond nutrient absorption

The primary function of the intestine lies in digestion of food and absorption of nutrients (Shanbhag and Tripathi, 2009; Lemaitre and Miguel-Aliaga, 2013). As this epithelium is continuously renewed, *Drosophila* researchers have paid more attention to the regulation of stem cells (Ohlstein and Spradling, 2006, 2007; Biteau et al., 2008; Lin et al., 2008; Takashima et al., 2008; Jiang et al., 2009; Scopelliti et al., 2014; Reiff et al., 2015; Brand et al., 2016; Hudry et al., 2016; Resnik-Docampo et al., 2017). Furthermore, many microbes reside in the intestine (Leulier and Royet, 2009; Shin et al., 2011; Buchon et al., 2013) and their activity can influence fly behavior and physiology (Sharon et al., 2010; Hang et al., 2014; Fischer et al., 2017; Leitão-Gonçalves et al., 2017; Mistry et al., 2017; Wong A. C. et al., 2017). One of the unique functions of the fly intestine is its role in copper (Filshie et al., 1971; Dubreuil, 2004; Burke et al., 2008) and iron (Tang and Zhou, 2013a; Rosas-Arellano et al., 2016) homeostasis. The specific roles of Fe-S and Moco enzymes in this tissue remain to be shown (Uhrigshardt et al., 2013).

### Secretory glands, fat bodies, and nephrocytes

The role of Fe-S proteins in secretory tissues, like the salivary glands and the fat bodies, and in hemolymph filtering tissues, like the Garland and pericardial nephrocytes is also not resolved. One common property of these tissues is that their nuclei undergo polyploidy (Nordman et al., 2011), hence nuclear Fe-S enzymes involved in DNA replication are expected to have an enhanced role.

### The nervous system

The ways in which iron and other metals relate to neurodegeneration have been reviewed (Zhu et al., 2014; Calap-Quintana et al., 2017), therefore a discussion on this topic will not be included here, except for the following points. Despite general agreement that *frataxin* is required for a functional nervous system, disagreement has been expressed on the cause, with different authors favoring oxidative stress (Llorens et al., 2007; Anderson et al., 2008; Kondapalli et al., 2008), iron toxicity (Soriano et al., 2013; Navarro et al., 2015), altered mitochondrial metabolism (Navarro et al., 2010; Tricoire et al., 2014; Calap-Quintana et al., 2015; Soriano et al., 2016), sphingolipid signaling (Chen et al., 2016b), and failure to maintain neuronal membrane potential (Shidara and Hollenbeck, 2010). We do not see any contradiction in the various positive claims made in the above-cited literature, whereas the negative claim that is often repeated—refuting a role for oxidative stress in explaining the phenotypes—normally arises because of failure to rescue the phenotypes with some transgenes as opposed to others. Similar failure could have various explanations: for example overexpression of Sod2 (Mockett et al., 1999) does not guarantee that the enzyme will be active in mitochondria with iron overload, at least our discussion of mitochondrial metabolism above (Figure 9) predicts otherwise. Furthermore, not all reactive oxygen species will act in the same way and their source and subcellular localization is also important to the effects they cause (Missirlis et al., 2003b). Last, cells handle iron in different ways: neurons and glia for example differ dramatically in their ability to store iron (Kosmidis et al., 2011, 2014) for reasons that are not understood. When ferritin mutants progress through early embryogenesis (they do so thanks to maternal contribution of iron-loaded ferritin to the oocyte) severe defects in the development of the nervous system ensue (González-Morales et al., 2015). Which step in brain development is most sensitive to the lack of ferritin has not been resolved. The blood-brain barrier regulates iron entry into the brain (Mehta et al., 2009), but we still do not know how iron traffics in the peripheral and central nervous systems or how Fe-S and/or Moco enzymes affect the circadian clock. Answers to questions of the basic cell biology of metal homeostasis are prerequisite for proposing better therapeutics when neuronal functions are compromised in disease (Zhu et al., 2014; Calap-Quintana et al., 2017; Ruland et al., 2017; Poetini et al., 2018).

## Conclusion

We cannot think of any biological function for which the fly will not require the biochemical participation of Fe-S clusters. We have attempted to describe our progress in understanding the role of Fe-S enzymes during the past 93 years since Otto Warburg firmly connected iron to respiration (Warburg, 1925) and also the Fe-S and Moco enzymes Xdh and Aox. We have used the fly as an example, but of course knowledge has been acquired from studying all forms of life as these enzymes are universal in character and may have formed at the origin of life (Hall et al., 1971; Russell and Martin, 2004; Nitschke and Russell, 2009; Schoepp-Cothenet et al., 2012; Varma et al., in review). We suggest that bioinorganic contributions to biology and bioenergetics be taken into account not only as having “house-keeping” roles, but also as an active component of the complex organization that characterizes all living systems (Frausto da Silva and Williams, 2001). Renewed attention on the inorganic chemistry underpinning *Drosophila* biology, together with the new analytical tools and methodologies available, should help integrate cellular metal homeostasis with metabolism (Dow, 2017; Navarro and Schneuwly, 2017). The humble fly has still much to contribute to our understanding of the workings of biology.

## Dedication

Dedicated to Stefan Grimm (1963–2014) who discovered, while working at Imperial College London, a moonlighting function for IkBα binding to the outer mitochondrial membrane: protecting cells from suicide.

## Author contributions

ZM: Performed the experiments on the mitochondrial Fe-S assembly core complex (Figure 3), determined the subcellular localization of fly Isd11 and Mocs3 (Figure 4), and performed the biochemical assays for aldehyde oxidase and sulfite oxidase (Figures 6, 7) during his Ph.D. thesis with SL; ZM, SL, and FM: Wrote the sections on Fe-S cluster assembly, Moco biosynthesis and the function of molybdoenzymes; FM: Found the quiescent mitochondria in the female gonad of larvae (Figure 10) and is responsible for the hypothesis that Sod2 acts as a mitochondrial iron sensor and other theories expressed in the latter part of the article; All authors have read, reviewed, and endorsed the full content of this publication.

### Conflict of interest statement

The authors declare that the research was conducted in the absence of any commercial or financial relationships that could be construed as a potential conflict of interest.
